# Dyke-Davidoff-Masson Syndrome: A Case Report

**DOI:** 10.7759/cureus.34868

**Published:** 2023-02-11

**Authors:** Venkata Anirudh Chunchu, Nishitha Kommalapati, Sai Sarath Kumar Pemma, Manish Prajwal Mane Manohar, Rahul Reddy Nalamalapu

**Affiliations:** 1 Medicine, Avalon University School of Medicine, Willemstad, CUW; 2 Medicine, John F. Kennedy University, Willemstad, CUW; 3 Surgery, Avalon University School of Medicine, Willemstad, CUW; 4 Radiology, Alluri Sitarama Raju Academy of Medical Sciences, Eluru, IND

**Keywords:** dyke-davidoff-masson syndrome (ddms), magnetic resonance imaging (mri), computed tomography (ct), hyperpneumatization, hemiplegia, cerebral hemiatrophy

## Abstract

Dyke-Davidoff-Masson syndrome (DDMS) is a rare neurological entity that is predominantly seen in childhood. Here, we present the case of a 13-year-old girl who was brought to the pediatric ward for general examination with a previous history of seizures, speech difficulty, facial deviation, and progressive left-sided hemiparesis that started at the age of two, followed by delayed developmental milestones. Computed tomography (CT) and magnetic resonance imaging (MRI) of the brain showed right cerebral hemiatrophy, ventriculomegaly, hyperpneumatization of the sinus, the decreased caliber of cortical veins, and skull thickening on the right were all characteristic findings of DDMS. Based on the history, clinical presentation, and imaging findings from CT and MRI, DDMS was confirmed. Identifying DDMS in a clinical setting can be challenging because of low awareness of the condition and varied clinical presentations. Although CT and MRI imaging are the gold standards in diagnosing DDMS, the early manifestations of the disease cannot be well-appreciated on a CT and would likely require an MRI. Since there is no standardized protocol for managing DDMS, the treatment is primarily symptomatic. Early identification and diagnosis of the syndrome are essential to aid the child’s mental and physical development through a multidisciplinary approach. There is also a need to improve awareness of DDMS so that the condition can be considered a potential differential diagnosis amongst other similar conditions and does not get misdiagnosed. The lack of a proper protocol for the management of DDMS prompts more research for a better understanding and early identification of the condition.

## Introduction

Dyke-Davidoff-Masson Syndrome (DDMS) was first described by Dyke, Davidoff, and Masson in 1933 [1]. It is characterized by hemiparesis, facial asymmetry, seizures, learning disabilities, and mental retardation. The etiology can be congenital or acquired. It is usually seen in childhood as a result of an intrauterine or early childhood alteration to the developing brain [2]. Typical radiographic findings include cerebral hemiatrophy with ipsilateral ventriculomegaly, compensatory enlargement of the skull, and significant sulcal spaces [3,4]. DDMS is relevant to the field of neurology and developmental pediatrics as it is a rare condition that has few documented cases in medical literature and can be easily misdiagnosed. DDMS can have a significant impact on a patient’s development and quality of life. Here, we present the case of a 13-year-old female who has a history of seizures, speech difficulty, facial deviation, and progressive left-sided hemiparesis who was diagnosed with DDMS from MRI and CT. The report provides detailed information on the patient’s medical history, physical examination, laboratory workups, and imaging findings. The report also highlights the characteristic clinical and radiographic findings of DDMS, as well as the possible causes and potential outcomes of the condition. This case report provides valuable information that can help other healthcare professionals in understanding and managing patients with DDMS.

## Case presentation

A 13-year-old female with a past history of seizures, speech difficulty, facial deviation, and progressive left-sided hemiparesis was brought to the pediatric ward for a general examination. The patient was born at full term to non-consanguineous parents with no significant history of antenatal or perinatal complications. The patient has reached all developmental milestones and appeared normal until 22 months. At age two, she had a fever for three days, followed by multiple episodes of generalized tonic-clonic seizures, each lasting up to 2-3 minutes, during which she experienced stiffening of the left upper extremity and deviation of the gaze upwards. She was admitted and treated at the local hospital and started on antiepileptic medication. She has had two seizure episodes since then, one at the age of three and the other at the age of four. Gradually, she developed weakness in her left upper and lower extremities along with facial deviation, followed by delayed developmental milestones, including fine motor and language delays. She could not copy a circle or a triangle and did not know her name or gender until she was eight years old. The following year, she lost her ability to speak and developed poor dexterity in her left hand, which is steadily deteriorating. Her parents reported that she did not have hearing or visual problems.

On physical examination, the patient is alert, oriented, and responsive to commands. Her intelligence quotient (IQ) was found to be 55, which corresponds to mild mental retardation. Vital signs: blood pressure was 110/72 mmHg, heart rate 82 beats per minute, respiratory rate 20 per minute, temperature 96℉, oxygen saturation 98%, and peripheral pulse 2+ bilaterally. She had a height and weight of 1.54 m and 63 kg, respectively, with a body mass index (BMI) of 26.6 kg/m², which is over the expected weight for her height and age. Vision and hearing are normal. Cranial nerve and sensory examinations were unremarkable. No neurocutaneous markers were found. The gait is abnormal. A neurological exam revealed 3/5 power in the left upper and lower extremities, brisk reflexes, and a flexor plantar response. Power at the left wrist joint was 2/5. The right upper and lower extremities had a score of 5/5. Hypotonia on the left upper and lower limbs was present. Upper and lower limb sensation and proprioception were normal. There was no neck rigidity, signs of cerebellar dysfunction, or bladder-bowel involvement. Other systemic examinations were unremarkable.

**Figure 1:**
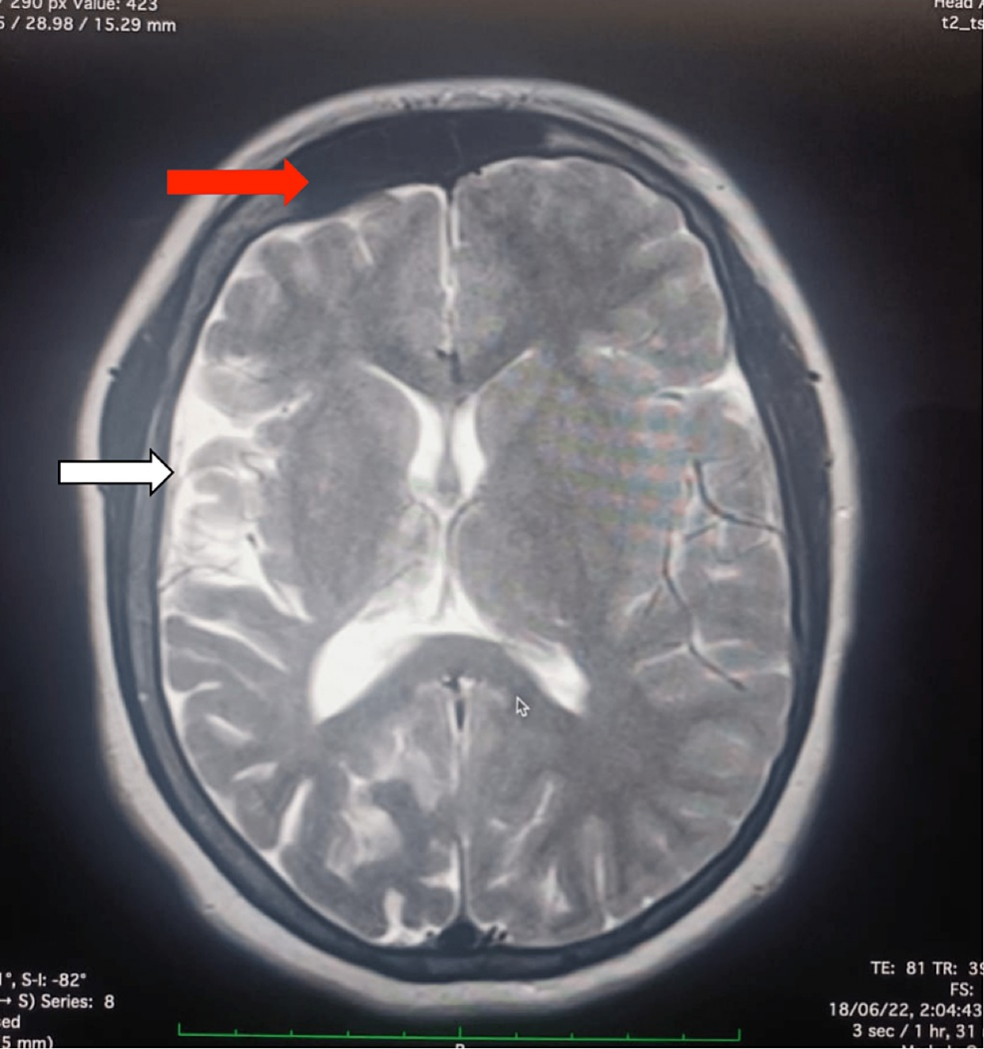
Axial T2-weighted MRI of the brain demonstrates hemiatrophy changes in the right cerebral hemisphere (white arrow) and hyperpneumatization of the frontal sinus (red arrow). MRI: magnetic resonance imaging

**Figure 2:**
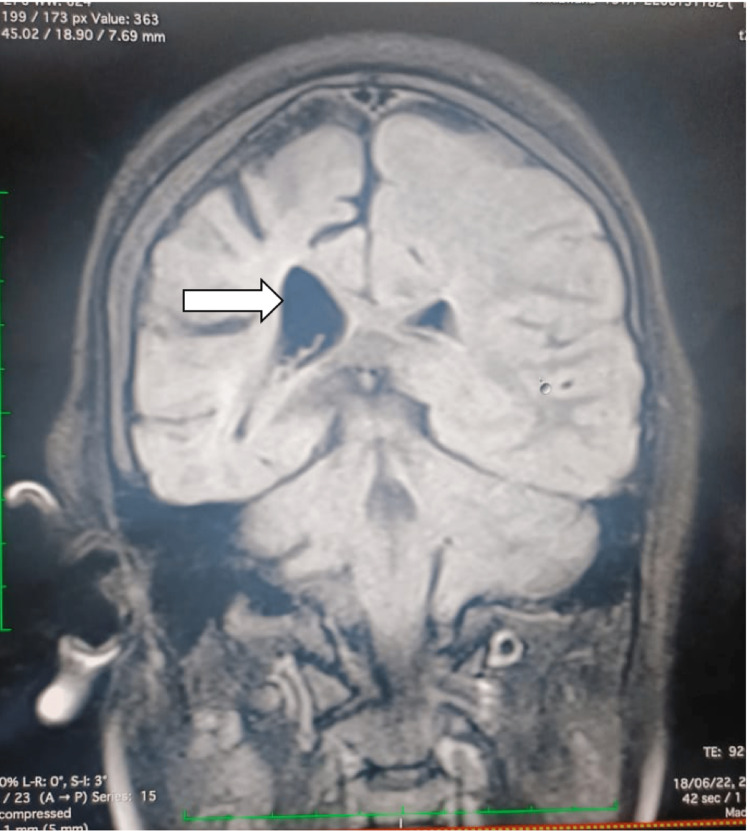
Coronal T2 FLAIR MRI of the brain demonstrates dilated lateral horn of right ventricle. MRI: magnetic resonance imaging; FLAIR: fluid-attenuated inversion recovery

**Figure 3:**
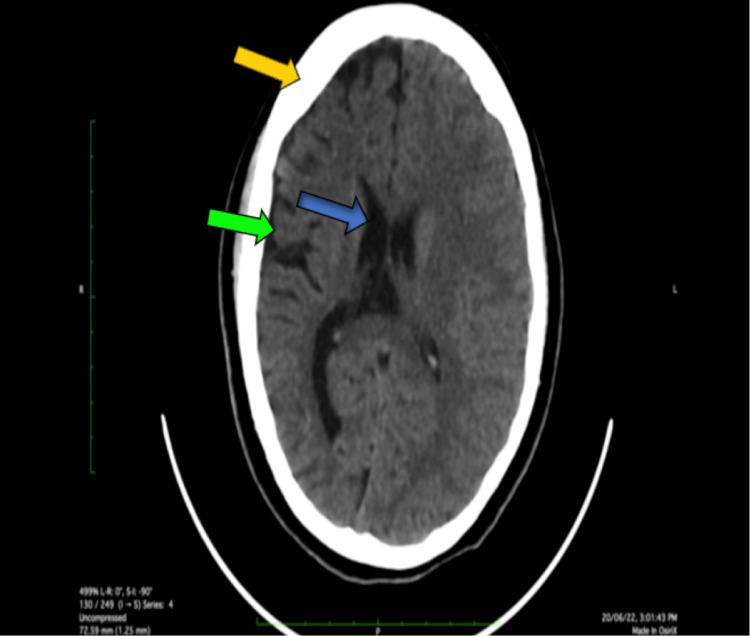
Plain CT images of the brain show atrophy of the right cerebral hemisphere (green arrow) with ipsilateral ventriculomegaly (blue arrow) and ipsilateral calvarial thickening (yellow arrow). CT: computed tomography

**Figure 4:**
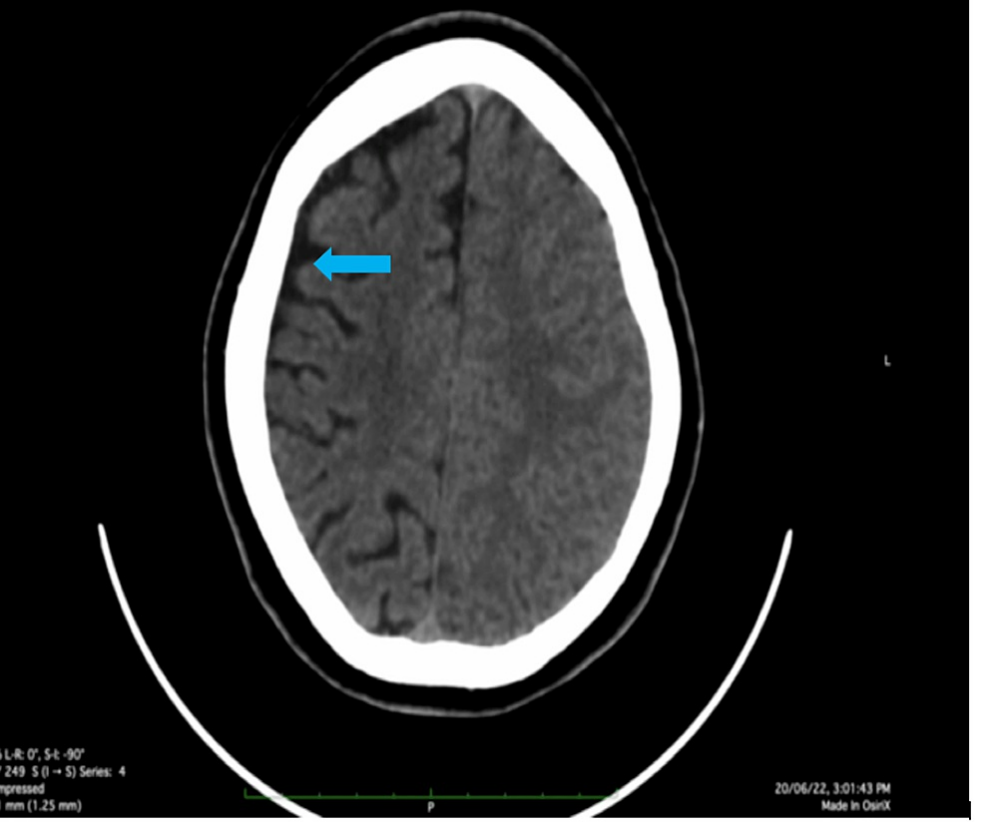
Plan CT images of the brain show widening of the sulci and atrophy of the gyri in the right cerebral hemisphere. CT: computed tomography

Laboratory workups include baseline investigations. Complete blood count and comprehensive metabolic panel investigations, such as renal, liver function, CRP, and electrolytes, were within normal limits. MRI of the brain revealed hemiatrophy changes in the right cerebral hemisphere with adjacent dilation of the ipsilateral ventricle and hyperpneumatization of the frontal sinus (Figure 1). In addition, a decreased caliber of cortical veins in the right cerebral hemisphere was noted. MRI of the brain revealed dilated lateral right ventricular horn (Figure 2). A plain and contrast CT of the brain was subsequently done, which revealed atrophy of the right cerebral hemisphere with dilatation of the ipsilateral ventricle and calvarial thickening noted in the right hemicranium (Figure 3). There is also a widening of sulci and atrophy of gyri on the right cerebral hemisphere (Figure 4). A final diagnosis of DDMS was made after comparing the imaging findings and clinical presentation.

Our patient was diagnosed with DDMS after carefully ruling out other neurological pathologies that can affect a similar pediatric age group and can present with similar symptoms. Imaging played a vital role in ruling out other disease conditions, especially pathologies like Rasmussen’s encephalitis and Sturge-Weber syndrome (SWS). The patient’s parents were educated about the syndrome and the disease’s possible progression. The patient was advised to have frequent neurological follow-ups to take care of any symptomatic changes or to consider other needs like physiotherapy in the future with disease progression.

## Discussion

DDMS was first defined in 1933 by Dyke, Davidoff, and Masson [1]. It is a rare neurological condition characterized by cerebral hemiatrophy or hypoplasia. The etiology can be congenital or acquired, which is caused by an injury to the developing brain during the prenatal period or in early childhood, usually traumatic [2]. Clinical features are variable and can be present with different manifestations according to the extent of brain injury. Patients typically present with clinical characteristics including facial asymmetry, recurrent seizures, contralateral hemiplegia, learning disability, mental retardation, and delayed developmental milestones. Occasionally, psychiatric disorders such as schizophrenia and schizoaffective disorder have also been reported. Cerebral hemiatrophy with ipsilateral ventriculomegaly, compensatory enlargement of the skull, and significant sulcal spaces are typical radiographic findings [3,4]. Other observed radiologic findings include hyperpneumatization of the frontal sinuses, ethmoid air cells, mastoid air cells, and air cells of the petrous temporal bone on the affected side [5-7]. They hypothesized that these were compensatory alterations caused by congenital or acquired brain injuries that reduced cerebral cortical volume.

The congenital form states that the cerebral insult likely occurred in-utero due to vascular malformation, cerebral infarction, coarctation of the mid-aortic arch, gestational vascular occlusion, and infections. In the acquired form, the cerebral insult likely occurred during the perinatal or postnatal period, caused by hypoxia, birth trauma, tumors, infections, prolonged febrile seizures, and intracranial hemorrhage [8]. In both sexes, either of the hemispheres can be affected by DDMS. However, the involvement of the male sex and the left hemisphere has been demonstrated to be more prevalent [9]. Identifying DDMS in a clinical setting can be challenging because of low awareness of the condition, and especially more so because of varied clinical presentations. Like in this patient where she initially presented only with seizures but then eventually, after a few years, displayed facial deviation, left-sided hemiparesis, speech difficulty, and mental deterioration. Any unsuspecting clinician would treat this patient only for the seizures and would fail to identify the larger cause, especially in underprivileged areas where it is difficult to obtain expensive imaging like MRI. Although CT and MRI imaging are the gold standards in diagnosing DDMS, the early manifestations of the disease cannot be well-appreciated on a CT and would likely require an MRI as it would provide more detail on cross-sectional images [2]. A thorough history, extensive neurological and cognitive assessment along with characteristic radiologic findings are needed to provide a correct diagnosis of DDMS.

Other differential diagnoses that should be differentiated from DDMS are Rasmussen encephalitis, SWS, Fishman syndrome, basal ganglia germinoma, linear nevus syndrome, and Silver-Russell syndrome, as these conditions share similar imaging findings and presentations as DDMS. Rasmussen encephalitis is a severe immune-mediated brain dysfunction in children with cognitive defects and seizures, with similar imaging findings as hemispheric atrophy, but calvarial changes are not observed [10]. SWS is distinguished by the classic triad of glaucoma, port-wine nevus, and leptomeningeal angiomas, which are the sources of stroke-like symptoms, seizures, hemiparesis, mental retardation, along with developmental delays [11]. Fishman syndrome is a rare congenital neurocutaneous disease characterized by intellectual disability, cerebral calcification, seizures, ipsilateral cerebral malformation, unilateral temporofrontal lipomatosis, and leptomeningeal lipomatosis [12]. Basal ganglia germinoma may present with cerebral hemiatrophy and progressive hemiparesis [3]. The characteristic features of linear nevus syndrome are facial nevus, cyclic refractory seizures, growth restriction, mental retardation, and unilateral ventriculomegaly [13]. Silver-Russell syndrome is an imprinting gene disorder characterized by severe intrauterine and postnatal growth restriction. Along with a severe delay in developmental phenotype that affects the height and bone length, it has a characteristic facial phenotype (triangular face, micrognathia with pointed chin, thin wide mouth, and broad forehead). It is also accompanied by additional dysmorphic features like fifth finger clinodactyly and hemihypertrophy without affecting mental capacity [14,15].

Since there is no standard protocol for managing DDMS, the treatment is primarily symptomatic, which includes anticonvulsants for seizures. In cases of intractable disabling seizures, hemispherectomy is an available neurosurgical option and has reported an 85% success rate. Long-term management includes physiotherapy for hemiparesis, occupational therapy, speech therapy for speech defects, psychiatric counseling, and medications if required [1,16].

## Conclusions

DDMS is a rare syndrome with few cases described in medical literature, and it can be easily misdiagnosed by inexperienced clinicians if not diligently looked after. Early identification and diagnosis of the syndrome are essential to aid the child’s mental and physical development through a multidisciplinary approach. Imaging studies play a crucial role in identifying such pathologies. Hence, although expensive, it is important to consider advanced imaging studies like MRI to facilitate the diagnosis and not stop after a CT to avoid any missed diagnosis of the disease. There is also a need to improve awareness of DDMS so that the condition can be considered a potential differential diagnosis amongst other similar conditions and does not get misdiagnosed. The lack of a proper protocol for the management of DDMS prompts more research and a better understanding of the condition.
